# Evaluation of oral nano-curcumin efficacy on respiratory function and quality of life in patients with bronchial non-atopic asthma: A randomized controlled trial

**DOI:** 10.22038/AJP.2023.22826

**Published:** 2024

**Authors:** Shahrzad Mohammadzadeh Lari, Amir Hooshang Mohamadpour, Davood Attaran, Mahmodreza Jafari, Omid Arasteh, Maryam Emadzadeh, Mahnaz Mozdourian, Soroush Attaran, Zahra Javidarabshahi

**Affiliations:** 1 *Lung Diseases Research Center* *, Mashhad University of Medical Sciences, Mashhad, Iran*; 2 *Department of Clinical Pharmacy, School of Pharmacy, Mashhad University of Medical Sciences, Mashhad, Iran*; 3 *Pharmaceutical Research Center, Pharmaceutical Technology Institute, Mashhad University of Medical Sciences, Mashhad, Iran*; 4 *Department of Pharmaceutics, School of Pharmacy, Mashhad University of Medical Sciences, Mashhad, Iran*; 5 *Clinical Research Development Unit, Ghaem Hospital, Mashhad University of Medical Sciences, Mashhad, Iran*

**Keywords:** Asthma, Nano-curcumin, Quality of life

## Abstract

**Objective:**

Asthma is a common disease and curcumin has modest effect in inflammatory disorders. This study investigated the efficacy of nano-curcumin on asthma.

**Materials and Methods:**

In this double-blinded randomized clinical trial, 60 patients with non-atopic bronchial asthma were randomly stratified in two groups of intervention (N=30) and control (N=30) groups. Apart from their standard treatment, the intervention group received 40 mg nano-curcumin (soft gel) three times daily while the control group received placebo. During the 60-day study, patients were assessed using spirometry to measure Forced expiratory volume in first second (FEV_1_). Asthma control test (ACT) was completed every 30 days and asthma quality of life questionnaire (AQLQ) was completed at the first and end of the study.

**Results:**

Totally, 31 patients (51.7%) were male and the mean age was 51.45±12.58 years. FEV_1 _was improved but there was no significant difference between intervention and control groups. ACT and AQLQ domains scores significantly improved. However, it was not statistically different between control and intervention groups.

**Conclusion:**

Nano-curcumin at administered dosage had no additive effect on the standard treatment in asthmatic patients.

## Introduction

Asthma is a common inflammatory lung disease affecting persons of different ages and ethnicity (Alhassan et al., 2016; Papi et al., 2018) cells become activated. Following the activation of mast cells and TH2 cells, production of inflammatory mediators such as different cytokines and chemokines will be induced. The most important inflammatory cytokines are Interleukin 4 (IL-4), IL-5, and Granulocyte macrophage colony-stimulating factor (GM-CSF) (Busse and Lemanske, 2001). It is proposed that most of these inflammatory cytokines and biomarkers are regulated by NF-КB pathway (Janssen-Heininger et al., 2009). The end result of this pathway is persistent inflammation (Busse and Lemanske, 2001). There are scoring systems defined for categorizing the severity of the exacerbations (Rodriguez et al., 2016).

Typically, treatment options of asthma are categorized into two groups including controller and reliever drugs. The most common controller drugs are corticosteroids and the most common reliever drugs are bronchodilators (Fanta, 2009). Because the main pathophysiologic cause of asthma is an inflammatory cascade, its main treatment is anti-inflammatory drugs. The most common anti-inflammatory drugs used in these patients are corticosteroids (Busse and Lemanske, 2001; Fanta, 2009). However, chronic use of systemic corticosteroids is associated with increasing adverse effects such as hyperglycemia, decreased immune function, osteoporosis, leg edema, ecchymosis, cushingoid features, skin disorders, and sleep disturbances (Yasir et al., 2020). On the other hand, asthma patients need to be treated for long-term. Therefore, in order to reduce corticosteroids adverse effects, inhalation forms of these drugs have been formulated (Fanta, 2009). Despite the many benefits of these formulation, they have disadvantages such as incorrect use by patients which can cause some adverse effects and inadequate drug-delivery (Rau, 2005; Ibrahim et al., 2015). Therefore, following incomplete drug-delivery and improper therapeutic response, noticeable patients with asthma remain symptomatic. Furthermore, it is possible that patient compliance to therapy is reduced. On the other hand, in spite of many different treatments for asthma, asthma remains a worldwide health problem. Therefore, the standard treatments of asthma are not always completely effective and additive therapeutic options can help to reduce the symptoms and inflammation of this disease. 

For ages, curcumin has been used against a variety of ailments including autoimmune diseases, metabolic diseases, cancer, cardiovascular diseases, liver diseases, and lung diseases (Mandal et al., 2020). Curcumin is an active ingredient of turmeric spices and has anti-inflammatory, and antioxidant properties (Shishodia et al., 2005; Mandal et al., 2020). Curcumin is safe in high doses for human subjects and only some modest side effects including diarrhea, rash, headache, and yellow stool have been reported (Hewlings and Kalman, 2017). However, some evidence proposed that chronic use of high doses of curcumin can act as a dose-dependent pro-oxidant (Yoshino et al., 2004). Thus, the best daily dose of curcumin is unclear. However, there is strong evidence showing that chronic use of curcumin up to about 150 mg/day is safe (Sharma et al., 2005). In spite of tolerability of high oral doses, bioavailability of oral use is low due to first pass metabolism, poor gastrointestinal absorption, and low aqueous solubility (Sharma et al., 2005; Mandal et al., 2020). To reach considerable bioavailability of curcumin, some strategies in drug formulation such as the use of nanoparticles were suggested. Curcumin poses an appropriate anti-inflammatory property, it does it by regulating the NF-κB, MAPK, AP-1, JAK/STAT and other signaling pathways, and inhibiting the production of inflammatory mediators (Peng et al., 2021).

Nanoparticle formulations can counter the hydrophobicity of curcumin (Mandal et al., 2020). Since the main pathophysiological pathway in asthma is inflammatory cascade and curcumin has anti-inflammatory effects, it may mitigate development of asthma through suppression of the NF-КB activity. As a result, it can play a role in controlling symptoms in patients with asthma as a possibility. 

In this study, we carried out a double-blinded randomized clinical trial to identify the effects of nanomicelle curcumin on improvement in FEV_1_ value, quality of life and control of symptoms in patients with non-atopic asthma disease. 

## Materials and Methods

**Study design**

This study is a multi-center double-blinded randomized clinical trial which was conducted in respiratory wards of Imam Reza and Qaem hospitals in Mashhad University of Medical Sciences. Sample size was estimated by specific clinical trial formula based on α= 0.05 and β= 0.8. Therefore, a sample size 30 patients was obtained for each group.

**Ethical considerations**

The ethical approval was obtained from Institutional Ethical Committee of Mashhad University of Medical Sciences (IR.MUMS.fm.REC.1395.259). This study was registered at the Iranian Registry of Clinical Trials (http://www.irct.ir) with the registration code IRCT20161226031584N2.

**Study population**

Patients with non-atopic bronchial asthma and age equal or more than 18 years old were included in our study. 

**Inclusion and exclusion criteria**

The exclusion criteria included: smoking, patients with other respiratory diseases, pregnancy, lactation, asthma exacerbation within last month, or having chronic diseases such as cardiac, renal or liver diseases. Written informed consent was taken from all of the included patients.

**Study protocol**

The patients were allocated into intervention and control groups based on simple randomization and random number table. In the intervention group, patients received 40 mg tablets of curcumin three times daily along with standard treatment of asthma (Gupta et al., 2013). In the control group, patients received placebo instead of curcumin tablets. Classification of asthma severity (mild, moderate, and severe, Table 1) and standard treatment was performed based on Global Initiative for Asthma (GINA) (Boulet et al., 2019) and National Asthma Education And Prevention Program (NAEPP) guidelines (National Heart Institute, 1997). Demographic information of patients was collected in special forms. Basic spirometry was performed for patients at the beginning of the study. Patients were visited every 15 days for 5 times. In order to evaluate the sign and symptoms of patients, the Asthma Control Test (ACT) was filled as a type of questionnaire-description every month for three times (Jia et al., 2013). The first time for ACT evaluation was at the beginning of the study. ACT is a patient self-administered questionnaire for recognizing asthma control. It includes five items, with 4-weeks recall (on daily symptoms and functioning) and scores based on a 5-point scale (scores range 5-25; score ≤15, poorly controlled asthma; 16–19, partly controlled asthma; and 20–25, well-controlled asthma, see supplementary Table 1 ) (Sigari et al., 2011).

Asthma Quality of Life Questionnaire (AQLQ) was filled to evaluate quality of life for patients at the beginning and end of the study (Grammatopoulou et al., 2008). Asthma Quality of Life Questionnaire (AQLQ) is a disease-specific health-related quality of life instrument consisting 4 domains [Symptoms (11 items), Activity Limitation (12 items), Emotional Function (5 items), and Environmental Exposure (4 items); scores range 1-7, with higher scores indicating better quality of life] (Miri et al., 2007). 

**Table 1:** Severity classification of asthma according to NAEPP guideline*

**Components of severity**		**Classification of asthma severity (Youths > 12 years of age and adults) **			
		**intermittent**	**Persistent**		
			**mild**	**moderate**	**Severe**
Impairment Normal FEV_1_/FVC: 8-19 y: 85% 20-39 y: 80% 40-59 y: 75% 60-80 y: 70%	symptoms	≤2 days/week	>2 days/week but not daily	Daily	Throughout the day
	Nighttime awakenings	≤ 2×/month	3-4×/month	>1×/week but not nightly	Often 7×/week
	Short-acting beta 2 agonist use for symptom control (not prevention of EIB)	≤2 days/week	≥2 days/week but not >1×/day	Daily	Several times per day
	Interference with normal activity	none	Minor limitation	Some limitation	Extremely limited
	Lung function	Normal FEV_1_ between exacerbation FEV_1_>80% predicted FEV_1_/FVC normal	FEV_1_≥80% predicted FEV_1_/FVC normal	FEV_1_>60% but <80% predicted FEV_1_/FVC reduced 5%	FEV_1_<60% predicted FEV_1_/FVC reduced >5%
Risk	Exacerbations requiring oral systemic corticosteroids	0-1/year	≥2/year		
		Consider severity and interval since last exacerbations. Frequency and severity may fluctuate over time for patients in any severity category			
		Relative annual risk of exacerbations may be related to FEV_1_			

* National Asthma Education And Prevention Program (NAEPP) guidelines (National Heart Institute, 1997).

**Study outcomes**

The primary endpoint of this study was increasing in FEV_1_ and secondary endpoint was improvement in control of symptoms and improving quality of life.

**Statistical method**

After data gathering, we used SPSS software version 22 for data analysis. In order to evaluate distribution normality, Kolmogorov-Smirnov test was used. We performed Chi-square or Fisher’s exact test for analysis of nominal data and Independent Samples T-test or Mann-Whitney test for categorical data. Paired samples T-test was performed to compare the quantitative variables before and after the intervention. Repeated measures ANOVA was used to compare the quantitative variables in the intervals of time between and within groups during our study. A p-value less than 0.05 was considered a statistical significance.

## Results

**Demographic findings**

In this double-blinded randomized clinical trial 150 patients were assessed for eligibility criteria and finally 60 patients, meeting the inclusion criteria were included. The patients were allocated to control and intervention groups in equal numbers. In general, 31 patients (51.7 %) were male, the range of age was 30-75-years old, and mean age of patients in this study was 51.45±12.58 years old. There was significant difference in age and weight between control and intervention groups. As shown in Table 2, the differences in other demographic characteristics and asthma severity between the two groups were not statistically significant.

**Figure 1:**
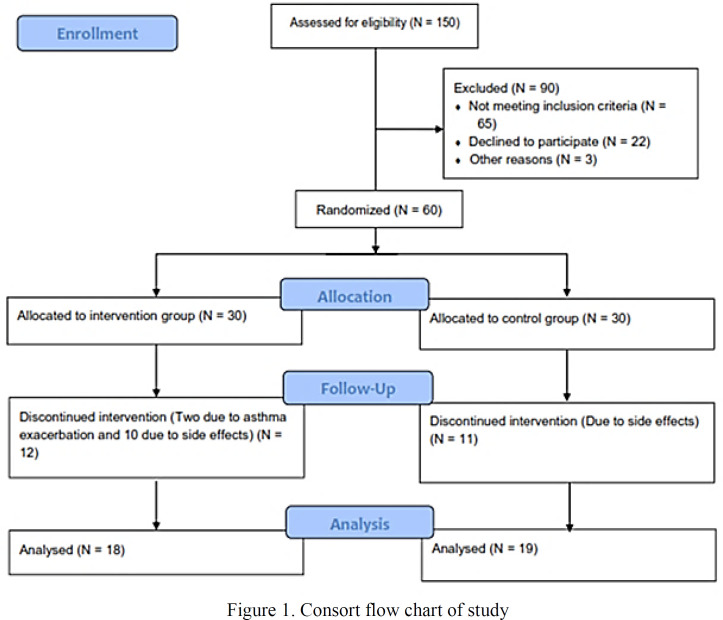
Consort flow chart of study

In our study, 23 patients were excluded due to adverse effects and exacerbation of asthma. Eighteen patients in the intervention group and 19 patients in the control group completed the study. Finally, 37 patients were analyzed (Figure 1).

**Table 2:** Demographic characteristics of the control and intervention groups

Characteristic		Intervention group (N=30)	Control group (N=30)	p-value
Age (year)		47.86±12.11	55.03±12.21	**0.026** ^*^
Sex (male %)		14 (45.2%)	17 (54.8%)	**0.438** ^***^
Weight (kg)		69.23±13.67	79.00±21.47	**0.041** ^*^
Height (cm)		161.30±8.27	165.53±8.77	**0.060** ^*^
BMI (kg/m^2^)		26.83±6.29	28.68±6.68	**0.273** ^*^
FEV_1_(%)		61.74±19.81	66.06±15.36	0.350*
ACT score		12.36±5.39	12.70±5.83	0.853**
AQLQ score		5.02±0.59	5.02±0.55	0.996*
Asthma severity	**Mild** **Moderate** **Severe** **Very severe**	6(20%) 9(30%) 15(50%) 0(0%)	0.284***	

*Independent samples T-test. ** Mann-Whitney test. ***Chi-square test

BMI: Body Mass Index

ACT: Asthma control test

**Baseline characteristics (primary outcome) comparison between the groups**

**Figure 2:**
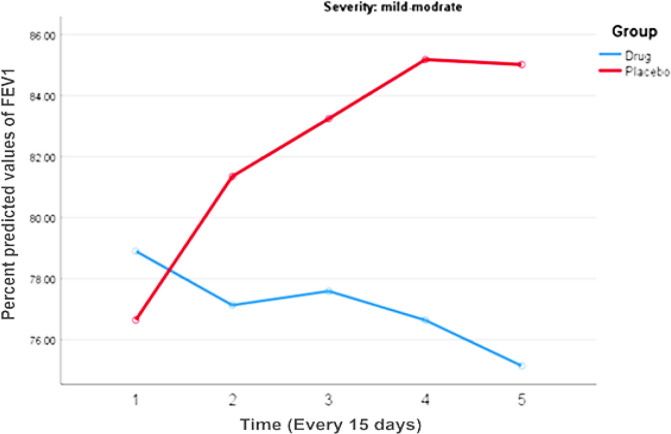
The comparison of the percent predicted values of FEV_1_ during the study period in mild-moderate asthma patients

**Figure 3:**
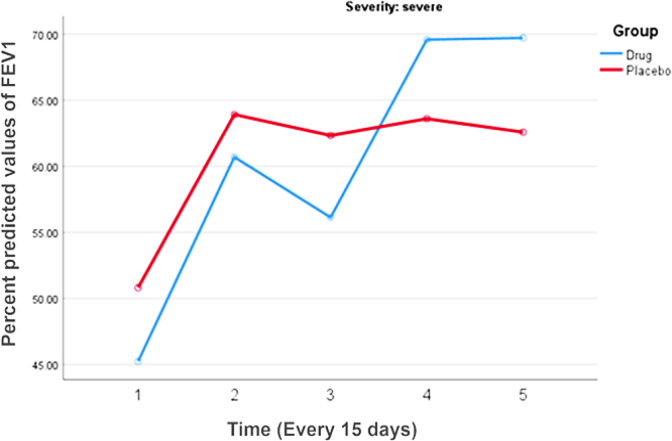
The comparison of the percent predicted values of FEV_1_ during the study period in severe asthma patients

At the beginning of study, there was no significant difference between the two groups in baseline values of FEV_1_ (p-value= 0.350), ACT score (p-value =0.853) or AQLQ score (p-value= 0.996). In addition, the distribution of asthma severity was not significantly different between the two groups at the start of the study (p = 0.284). Considering the increasing trend of FEV_1_, this value was not significantly different between the two groups in any of the included patients (p = 0.401, Table 3). Congruent results have been seen in sub-group patients with mild to moderate asthma (p = 0.394, Table 4, Figure 2). The trend of increasing of percent predicted FEV_1_ in severe asthma patients was not significant between the two groups (p = 0.968, Table 5, Figure 3), but this trend was more considerable in the intervention group.

**Table 3:** The comparison of the percent predicted values of FEV_1_ during the study period in all asthmatic patients between two groups*

FEV_1_ (% predicted)	Intervention group	Control group	p-value between two groups
Visit 1	63.93±21.30	67.52±14.73	0.45
Visit 2	69.82±24.08	75.20±15.90	0.31
Visit 3	68.05±21.97	75.86±16.75	0.12
Visit 4	73.50±24.31	77.56±16.00	0.44
Visit 5	72.72±24.30	77.10±14.70	0.40
p-value	**0.222**	**0.001**	**0.401**

*Repeated measures ANOVA. Visit 1: at the beginning of the study (Day 1), Visit 2: Day 15, Visit 3: Day 30, Visit 4: Day 45, and Visit 5: Day 60*.*

**Table 4:** The comparison of the percent predicted values of FEV_1_ during the study period in mild to moderate asthma patients*

FEV1 (% predicted)	Intervention group	Control group	p-value between two groups
Visit 1	78.90±12.34	76.64±8.23	0.40
Visit 2	77.13±24.27	81.35±10.42	0.38
Visit 3	77.59±21.20	83.24±6.77	0.16
Visit 4	76.64±21.04	85.18±10.27	0.05
Visit 5	75.14±21.30	85.01±6.69	0.01
P-value	**0.912**	**0.042**	**0.394**

*Repeated measures ANOVA. Visit 1: at the beginning of the study (Day 1), Visit 2: Day 15, Visit 3: Day 30, Visit 4: Day 45, and Visit 5: Day 60.

**Table 5:** The comparison of the percent predicted values of FEV_1_ during the study period in severe asthma patients^*^

FEV1 (% predicted)	Intervention group	Control group	p-value between two groups
Visit 1	45.23±13.69	50.81±6.37	0.04
Visit 2	60.70±21.89	63.91±18.87	0.54
Visit 3	56.13±17.39	62.33±21.61	0.22
Visit 4	69.58±28.88	63.60±15.70	0.32
Visit 5	69.71±28.86	62.58±14.28	0.23
P-value	**0.066**	**0.033**	**0.968**

*Repeated measures ANOVA. Visit 1: at the beginning of the study (Day 1), Visit 2: Day 15, Visit 3: Day 30, Visit 4: Day 45, Visit 5: Day 60.

**Comparison of secondary outcomes after intervention between the groups**

Assessment of ACT questionnaire scores showed that overall asthma control was significantly increased in all included patients (p <0.001, Table 6). In the discriminant analysis, each of the intervention and control group also had significant changes in ACT score during the study (in each group p <0.001). But this trend did not show significant difference between the two groups (p = 0.354).

As shown in Table 7, our results showed that the scores of all AQLQ domains which show patient’s quality of life were significantly higher in the second visit compared to the first visit in both the intervention and control groups. In each of the AQLQ score, there was no significant difference between the two groups at either the first (before the start of the study) or the second time (at the end of the study). 

We also found that FEV_1_ percentage variation after bronchodilator administration between the two study groups was not significantly differenced (p =0.986, Table 8) that means nano-curcuminin did not alter the response to bronchodilator in asthmatic patients.

Regarding the adverse effects due to curcumin, no significant difference was observed between the two groups (p >0.99). The most reported adverse effect among patients was gastritis which was observed in 9 and 6 patients in the control and intervention groups respectively (Table 9).

**Table 6:** Comparison of ACT scores between the control and intervention group^*^

ACT score	Intervention group	Control group	p-value between two groups
Visit 1	12.55 ± 5.13	11.58 ± 5.39	0.47
Visit 2	19.38 ± 3.64	18.41 ± 3.24	0.28
Visit 3	24.61 ± 0.84	23.76 ± 1.52	0.009
p-value	**< 0.001**	**< 0.001**	**0.354**

*Repeated measures ANOVA. Visit 1: at the beginning of the study (Day 1), Visit 2: Day 30, and Visit 3: Day 60.

**Table 7:** Comparison of Asthma Quality of Life Questionnaire (AQLQ) between the two study groups

Domain of AQLQ		Intervention group	Control group	p between two groups*
symptoms	Visit 1	4.64±0.90 6.23±0.50	4.73±1.00 5.87±0.65	**0.750** **0.79**
	Visit 2			
Statistical differences between two visits **		**<0.001**	**<0.001**	
Environmental	Visit 1	5.23±0.70 5.58±0.44	4.84±0.67 5.40±0.60	**0.457** **0.311**
	Visit 2			
Statistical differences between two visits **		**0.016**	**<0.001**	
emotional	Visit 1	6.16±0.6 5.63±0.48	5.19±0.66 5.74±0.45	**0.840** **0.502**
	Visit 2			
Statistical differences between two visits **		**0.001**	**<0.001**	
activity	Visit 1	5.24±0.61 6.07±0.32	5.00±0.78 5.77±0.55	**0.851** **0.061**
	Visit 2			
Statistical differences between two visits **		**<0.001**	**<0.001**	
Total score	Visit 1	5.05±0.50 5.90±0.36	4.88±0.54 5.65±0.41	**0.996** **0.064**
	Visit 2			
Statistical differences between two visits **		**<0.001**	**<0.001**	

*Independent samples T-test. ** Paired Samples T-test. Visit 1: at the beginning of the study (Day 1), and Visit 2: Day 60.

**Table 8:** Comparison of FEV_1_ percentage variation after bronchodilator administration between the two study groups*

FEV_1_ (% perdicted)	Intervention group	Control group	p between two groups
Visit 1	5.31±8.45	5.54±6.15	0.90
Visit 2	7.06±11.897	5.27±5.53	0.45
Visit 3	7.33±8.05	5.98±8.78	0.53
Visit 4	3.14±3.58	5.28±6.49	0.11
Visit 5	5±7.70	5.29±7.01	0.87
p-value	**0.87**	**0.99**	** 0.986**

*Repeated measures ANOVA. Visit 1: at the beginning of the study (Day 1), Visit 2: Day 15, Visit 3: Day 30, Visit 4: Day 45, Visit 5: Day 60.

**Table 9:** Comparison of adverse reactions between the two study groups*

		**Intervention group**	**Control group**	**p between two groups**
**Adverse reactions**	gastritis	6(20%)	9(30%)	**0.999**
	pruritus	1(3.3%)	2(6.7%)	
	urticaria	1(3.3%)	-	
	Gastroesophageal reflux	2(6.7%)	-	
	Bleeding tendency	1(3.3%)	-	
**Without adverse reactions**		19(63.3%)	19(63.3%)	

* Chi-square test

## Discussion

The results of FEV_1_ changes showed increased FEV_1_ values in all, mild to moderate and severe asthmatic patients, which was more pronounced in the control, compared to the intervention group. Probably, regular follow-up is the reason of FEV1 improvement in all patients. 

Only percent predicted FEV_1_ values in the severe asthmatic patients in the intervention group were more increased than the control group suggesting a possible therapeutic effect of curcumin in severe asthmatic patients. In addition, the FEV_1_ results indicate the absence of the effect of curcumin treatment on FEV_1_ value during the study period. However, ACT and AQlQ domain scores significantly increased in the intervention group as like as control group. As result, respiratory scores enhancement during the study period indicated increased control of disease. The reason of these findings is not known to us but could be due to qualitative notion of the respiratory symptoms.

Asthma is primarily an inflammatory airway disease (Mims, 2015). So, the cornerstone treatment of asthma is anti-inflammatory or controller drugs (Fanta, 2009). There are a wide variety of controller drugs for the treatment of asthma, however, a great deal of patients remains symptomatic with these therapeutic options. Therefore, in many patients with poorly responsive asthma, add-on therapeutic options can be used. Recently, some studies have evaluated the effects of additive therapy to the standard treatment in patients with asthma. For many years, herbal medicine has been used in different diseases. 

There are some studies about the efficacy of curcumin on inhibition of airway inflammation in pre-clinical and clinical studies, and the results of most of them showed that curcumin could help to prevent airway inflammation through the inhibition of NF-КB and decrease in inflammatory biomarkers. However, the human studies are scarce (Ram et al., 2003; Epstein et al., 2010).

Kim et al. achieved similar results to our study (Kim et al., 2011). In this pilot study, curcumin did not offer any clinically significant advantages. The number of patients in treatment and control group were 9 and 6 respectively. The duration of the study was over six-month period on patients with atopic asthma. The number of included patients and duration of follow up were not similar to ours. In addition, the formulation of curcumin which was administered differed from our formulation. 

In other studies, curcumin has shown different results in patients with asthma than the present study. In one study which was conducted in Bosnia (Jusufovic, 2017), the administration of curcumin was associated with improvement in FEV_1_ value, ACT score, and AQLQ score. 

The duration of this study was similar to our study but the sample size was larger. In addition, higher doses of curcumin (1000 mg) was administrated in above-mentioned study. However, in our study the dose of nano-curcumin was 40 mg 3 times daily. The findings of our study run contrary to this research. Contrary to our study, the standard treatment was not used in the control group. Another difference was the type of curcumin formulation which was used. We used the nanomicelle curcumin formulation. Additionally, mild, moderate and severe asthmatic patients were included in our study while they included only moderate asthmatic patients. 

Also in Abidi et al. (Abidi et al., 2014) study in 60 patients with mild to moderate bronchial asthma, curcumin administration was associated with improvement in FEV_1_ and hematologic parameters. But there was no improvement in the asthma symptoms. The duration of this study was less than our study (30 days). In contrast to our study, the administered dose of curcumin in this study was higher (1000 mg daily) and the formulation was not similar. The mean age of patients in our study was higher than this study (50 versus 31-years-old). Hence, elderly patients may have lower medication adherence. Besides, in our study, FEV_1_ values in the intervention group during the study was not declined.

On the other hand, obesity is typically associated with poor control of asthma patients. Although the patients in our study was not categorized according to weight, but the mean body mass index (BMI) is higher than aforementioned study (27.75 *vs.* 22.88). Finally, although the FEV_1_ value was improved in this study, the improvement in patient's symptoms, which was more important in quality of life, had not been changed. 

In our study, further statistical analyses were done for better comparison based on disease severity. All asthmatic patients were divided into two subgroups: mild-moderate and severe asthma. In mild-moderate patients, we did not observe any significant difference between the intervention and control group. Increasing of FEV_1_ in severe asthmatic patients was higher than control group.

Additional strengths of this study included the use of standard questionnaires along with FEV_1_ assessment. ACT and AQLQ domain scores significantly improved in both intervention and control groups. Probably, increasing FEV_1_ (significant even non-significant) has been the reason of symptoms and quality of life improvement.

Besides the potential benefits of nanomicelle curcumin as a nano-formulated drug, attention is also drawn to the questions how we should manage the adverse effects due to the nanoparticle formulations. There is evidence which showed that some special nanoformulation drugs were associated with pseudo allergic response (Brand et al., 2017). Therefore, there is some doubt in our research whether nanoformulation had an extra side effect in patients who received nano-curcumin.

**The study limitations**

There were some limitations in our study. At first, the sample size of this study was not enough. In addition, the duration of follow up was not long enough. In Zeng et al., it has been demonstrated in pre-clinical study that curcumin has an inhibitory effect on the proliferation of airway smooth muscle cells (Zeng et al., 2013). Thus, the long-term administration of curcumin is needed to prevent hyperplasia in airways. 

So, future studies with larger sample size and longer duration of follow up would be associated with more reliable results. If future studies confirm the beneficial effects of curcumin in asthmatic patients, it potentially improves treatment adherence considering the easy administration. Therefore, it would be a logical choice in these patients.

The results of the present study only suggest a therapeutic effect of nano-curcumin in severe asthmatic patient based on non-significant increase in FEV_1 _predicted values. Therefore, further studies with higher sample size, longer treatment period and different curcumin doses in asthmatic patients with different severity special more severe patients should be performed in the future.

## Conflicts of interest

The authors have declared that there is no conflict of interest.

## Supplementary

**Table S1:** Asthma Control Test (ACT) scores

**Question 1** In the past 4 weeks, how much of the time did your asthma keep you from getting as much done at work, school or at home?				
All of the time 1	Most of the time 2	Some of the time 3	A little of the time 4	None of the time 5
**Question 2** During the past 4 weeks, how often have you had shortness of breath?				
More than once a day 1	Once a day 2	3-6 times a week 3	Once or twice a week 4	Not at all 5
**Question 3** During the past 4 weeks, how often did your asthma symptoms (wheezing, coughing, shortness of breath, chest tightness or pain) wake you up at night or earlier than usual in the morning?				
4 or more nights a week 1	2 -3 nights a week 2	Once a week 3	Once or Twice 4	Not at all 5
**Question 4** During the past 4 weeks, how often have you used your rescue inhaler or nebulizer medication (such as Salbutamol)?				
3 or more times per day 1	1-2 times per day 2	2-3 times per week 3	Once a week or less 4	Not at all 5
**Question 5** How would you rate your asthma control during the past 4 weeks?				
Not Controlled at all 1	Poorly Controlled 2	Somewhat Controlled 3	Well Controlled 4	Completely Controlled 5

## References

[B1] Abidi A, Gupta S, Agarwal M, Bhalla HL, Saluja M (2014). Evaluation of efficacy of curcumin as an add-on therapy in patients of bronchial asthma. J Clin Diagn Res.

[B2] Alhassan S, Hattab Y, Bajwa O, Bihler E, Singh AC (2016). Asthma. Crit Care Nurs Q.

[B3] Arbes SJ (2012). Do all asthmatics with atopy have atopic asthma?. J Allergy Clin Immunol.

[B4] Boulet L-P, Reddel HK, Bateman E, Pedersen S, FitzGerald JM, O'Byrne PM (2019). The global initiative for asthma (GINA): 25 years later. Eur Respir J.

[B5] Brand W, Noorlander CW, Giannakou C, De Jong WH, Kooi MW, Park MV, Vandebriel RJ, Bosselaers IE, Scholl JH, Geertsma RE (2017). Nanomedicinal products: a survey on specific toxicity and side effects. Int J Nanomedicine.

[B6] Busse WW, Lemanske RF (2001). Asthma. N Engl J Med.

[B7] Epstein J, Sanderson IR, Macdonald TT (2010). Curcumin as a therapeutic agent: the evidence from in vitro, animal and human studies. Br J Nutr.

[B8] Fanta CH (2009). Asthma. N Engl J Med.

[B9] Grammatopoulou E, Skordilis E, Koutsouki D, Baltopoulos G (2008). An 18-item standardized asthma quality of life questionnaire-AQLQ(S). Qual Life Res.

[B10] Hewlings SJ, Kalman DS (2017). Curcumin: a review of its' effects on human health. Foods.

[B11] Ibrahim M, Verma R, Garcia-Contreras L (2015). Inhalation drug delivery devices: technology update. Med Devices (Auckl).

[B12] Janssen-Heininger YM, Poynter ME, Aesif SW, Pantano C, Ather JL, Reynaert NL, Ckless K, Anathy V, van der Velden J, Irvin CG, van der Vliet A (2009). Nuclear factor kappaB, airway epithelium, and asthma: avenues for redox control. Proc Am Thorac Soc.

[B13] Jia CE, Zhang HP, Lv Y, Liang R, Jiang YQ, Powell H, Fu JJ, Wang L, Gibson PG, Wang G (2013). The asthma control test and asthma control questionnaire for assessing asthma control: Systematic review and meta-analysis. J Allergy Clin Immunol.

[B14] Juniper EF (1998). Effect of asthma on quality of life. Can Respir J.

[B15] Jusufovic EKM, Jusufovic A, Becarevic M, Al-Ahmad M, Nurkic J, Osmic M, Nadarevic A, Petrak F, Halilovic D, Sejdinovic R, Prnjavorac B (2017). Curcumin as an add-on therapy of moderate partially controlled asthma. Eur Respir J.

[B16] Kim DH, Phillips JF, Lockey RF (2011). Oral curcumin supplementation in patients with atopic asthma. Allergy Rhinol (Providence).

[B17] Mandal M, Jaiswal P, Mishra A (2020). Role of curcumin and its nanoformulations in neurotherapeutics: A comprehensive review. J Biochem Mol Toxicol.

[B18] Masoli M, Fabian D, Holt S, Beasley R (2004). The global burden of asthma: executive summary of the GINA Dissemination Committee report. Allergy.

[B19] Mims JW (2015). Asthma: definitions and pathophysiology. Int Forum Allergy Rhinol.

[B20] Miri S, Montazeri A, Heidarnazhad H (2007). Measurement of quality of life in Iranian adult patients with asthma, translation and validation of the Persian version of the asthma quality of life questionnaire (AQLQ). Ann Allergy Asthma Immunol.

[B21] National Heart L, Institute B (1997). National asthma education and prevention program (NAEPP). Expert panel report.

[B22] Nunes C, Pereira AM, Morais-Almeida M (2017). Asthma costs and social impact. Asthma Res Pract.

[B23] Papi A, Brightling C, Pedersen SE, Reddel HK (2018). Asthma. Lancet.

[B24] Ram A, Das M, Ghosh B (2003). Curcumin attenuates allergen-induced airway hyperresponsiveness in sensitized guinea pigs. Biol Pharm Bull.

[B25] Rau JL (2005). The inhalation of drugs: advantages and problems. Respir Care.

[B26] Sharma RA, Gescher AJ, Steward WP (2005). Curcumin: the story so far. Eur J Cancer.

[B27] Shishodia S, Sethi G, Aggarwal BB (2005). Curcumin: getting back to the roots. Ann N Y Acad Sci.

[B28] Sigari N, Sigari N, Ghasri H, Rahimi E, Mohammadi S (2011). Validation of Persian version of asthma control test based on new Global Initiative for Asthma Guidelines. Tanaffos.

[B29] Yasir M, Goyal A, Bansal P, Sonthalia S (2020). Corticosteroid Adverse Effects. StatPearls.

[B30] Yoshino M, Haneda M, Naruse M, Htay HH, Tsubouchi R, Qiao SL, Li WH, Murakami K, Yokochi T (2004). Prooxidant activity of curcumin: copper-dependent formation of 8-hydroxy-2'-deoxyguanosine in DNA and induction of apoptotic cell death. Toxicol In Vitro.

[B31] Zeng X, Cheng Y, Qu Y, Xu J, Han Z, Zhang T (2013). Curcumin inhibits the proliferation of airway smooth muscle cells in vitro and in vivo. Int J Mol Med.

